# Systemic Administration and Targeted Delivery of Immunogenic Oncolytic Adenovirus Encapsulated in Extracellular Vesicles for Cancer Therapies

**DOI:** 10.3390/v10100558

**Published:** 2018-10-13

**Authors:** Mariangela Garofalo, Alessandro Villa, Nicoletta Rizzi, Lukasz Kuryk, Vincenzo Mazzaferro, Paolo Ciana

**Affiliations:** 1 Department of Oncology and Hemato-Oncology, Center of Excellence on Neurodegenerative Diseases, University of Milan, 20122 Milan, Italy; alessandromaria.villa@unimi.it (A.V.); vincenzo.mazzaferro@unimi.it (V.M.); 2 Center of Excellence on Neurodegenerative Diseases, University of Milan, 20133 Milan, Italy; nicoletta.rizzi@unimi.it; 3 Targovax Oy, Clinical Science, 00180 Helsinki, Finland; lukasz.kuryk@targovax.com; 4 Department of Virology, National Institute of Public Health—National Institute of Hygiene, 00-791 Warsaw, Poland; 5 Gastrointestinal Surgery and Liver Transplantation Unit, Fondazione IRCCS Istituto Nazionale dei Tumori, 20133 Milan, Italy

**Keywords:** Oncolytic adenovirus, extracellular vesicles, lung cancer, immunocompetent mouse model, in vivo imaging

## Abstract

Oncolytic viruses (OV) are engineered to infect, replicate in and kill cancer cells. Currently, the OV therapeutic approach is mainly restricted to neoplasia amenable to direct local administration of viral particles, while the possibility of a systemic delivery of cancer-tropic viruses would extend the OV application to the treatment of metastatic neoplasia. Herein, we applied *in vivo/ex vivo* imaging to demonstrate that cancer tropism is achieved when OV are encapsulated inside extracellular vesicles (EV) administered intravenously (i.v.), but not when injected intraperitoneally (i.p.). Moreover, we show that the therapeutic procedure adopted does not alter the immunomodulatory properties of the viruses.

## 1. Introduction

Oncolytic viruses (OV) are a new class of therapeutic agents promoting anti-tumor responses through a mechanism of action based on selective tumor cell killing and induction of systemic anti-tumor immunity [1,2]. Oncolytic virotherapy is emerging as a potential anti-cancer strategy since pre-clinical [3] and clinical trials have provided encouraging results in terms of safety and efficacy. Recently, the approval of the first oncolytic virus, Imlygic (T-Vec, talimogene laherparepvec), in the Western world, by the US Food and Drug Administration (FDA) and the European Medicines Agency (EMA), has opened up new hope for improved treatment of cancer.

OV are often administered locally; intra-tumorally (i.t.), or systemically; intraperitoneally (i.p.) and intravenously (i.v.). Although local administration should deliver all viral particles directly to the tumor, there are a few advantages associated with the systemic approach. Firstly, not all tumors are amenable to local administration because they may consist of several small nodules spread out over a large area, or they are in an anatomic location inaccessible by a direct approach (e.g., brain); indeed, systemic delivery has a higher chance of reaching metastases throughout the body as well as the primary lesion. Moreover, the ability of some OV to induce antitumor immune responses may be enhanced when administered systemically [4]. The systemic approach may be limited when using adenovirus by the presence of neutralizing antibodies that may increase the clearance and decrease the level of circulating particles [5,6]. Thus, for solid tumors and disseminated malignancies, an innovative procedure for a systemic and targeted delivery of OV is highly warranted. To enhance OV delivery to neoplastic tissue, several approaches have been developed, including: (i) viral capsid modification with the addition of polyethylene glycol (PEG) polymers to reduce immunogenicity and increase the circulation time in the blood; an approach that, however, resulted also in a significant reduction of the viral infectivity [5,7]; (ii) the use of drug carrier systems, such as liposomes [8,9], to increase the tumor-specific transduction of OV; a strategy that demonstrated poor serum stability of the particles and induced immune rejections in the host, thus preventing its further development [10]; (iii) recent studies reported that OV can be selectively delivered into neoplastic cells by tumor microparticles for virotherapy [11].

Natural carriers such as red blood cells, cancer cells, dendritic cells, stem cells, viruses and bacteria have been used as drug carriers [12,13]. Interestingly, mesenchymal stem cells have been used as a delivery system because of their ability to release drugs, through their exosomes, directly to tumor cells [14]. Several works demonstrated that extracellular vesicles (EVs) themselves could be purified and used as drug delivery vehicles. EVs are nanometer- to micron-sized lipid membrane-bound vesicles that are able to physiologically transfer biological molecules from one cell to another over long range distances within the body [15]. EVs have the ability to carry a variety of macromolecules such as DNA, RNA, proteins, and small molecules, such as doxorubicin, curcumin, or paclitaxel [16], and can be used as effective, targeted, and non-immunogenic drug carriers [17,18].

Although it has been reported that EVs may participate in tumor development, and in the acquisition of the cancer hallmark capabilities affecting angiogenesis, tumor proliferation, invasion, and metastasis [19,20], their safety profile has been studied in a phase I clinical trial [21]. A number of clinical trials based on the use of EVs have been reported [22], including a trial for the development of cancer vaccines that used dendritic cell-derived EVs [23] for the treatment of metastatic melanoma [24] and advanced non-small cell lung cancer patients [21]. In a previous work, we have reported that cancer cell-derived EVs could be useful vehicles for systemic delivery of OV and paclitaxel for the treatment of lung cancer [25]. In this study, we tested the administration of the EV-encapsulated Ad5D24 OV by two systemic routes, i.p. and i.v. For these experiments we used a fluorescent dye [26] as a tracking agent and *in vivo/ex vivo* imaging technologies as a detection system for the characterization of the whole-body biodistribution of this OV formulation. Our results demonstrated a clear tumor-selective delivery of the oncolytic virus encapsulated in EVs when administered i.v.; a tropism that was not observed with the i.p. route of administration.

## 2. Materials and Methods

### 2.1. Cell Line, Virus

LL/2 mouse lung cancer cell line was purchased from the American Type Culture Collection (ATCC, Manassas, Virginia, USA). The cells were cultured at 37 °C and 5% CO_2_ in Dulbecco’s modified eagle medium (DMEM, Lonza, Basel, Switzerland) supplemented with 10% fetal bovine serum (FBS, Gibco Laboratories, Waltham, MA, USA), 1% of 100 u/mL penicillin/streptomycin (Gibco Laboratories) and 1% l-glutamine (Gibco Laboratories). Oncolytic adenovirus Ad5D24 was characterized by performing titration (VP/mL) and molecular analyses (PCR, restriction enzyme assay) to check virus genome stability and integrity as described elsewhere [27], expanded in human lung cancer cell line A549 and purified on cesium chloride gradients. The viral particle concentration was determined by OD_260_-reading and standard TCID_50_ (tissue culture infectious dose 50) assay was performed to determine infectious particle titer.

### 2.2. Production of Extracellular Vesicles (EV), EV-Virus and Lipophilic Dye Loaded EV Formulations

In order to produce EVs, 2.6 × 10^6^ LL/2 cells were plated into T-175 flask in medium supplemented with 5% FBS. The FBS growth media was ultra-centrifuged overnight (110,000× *g* at 4 °C for 18 hours, Optima LE-80K ultracentrifuge, rotor type 50.2, Beckman Coulter, Brea, CA, USA) to remove EVs present in serum. Cells were cultured at 37 °C and 5% CO_2_ until a cytopathic effect was seen, where upon the media was collected.

EVs were isolated from the conditioned medium using differential centrifugation steps. First the conditioned medium was centrifuged at 500× *g* in 4 °C for 10 minutes to pellet cells (Allegra X-15R Centrifuge, Beckman Coulter). Then, the supernatant was collected and ultra-centrifuged for 2 hours at 100,000× *g* in 4 °C, using Optima L-80 XP ultra-centrifuge (Beckman Coulter) with rotor SW32Ti (Beckman Coulter). The supernatant was aspirated and EV-containing pellets were re-suspended in PBS (Lonza) 100 μL and stored at −80 °C.

EV-encapsulated viruses (EV-Virus) were produced as previously described [25]. Virus samples were incubated in 100 mM NaOH at room temperature for 20 minutes in order to inactivate any free, not-EV-encapsulated virus present. Free viruses used as control were always inactivated for each experiment performed as previously reported [25]. Samples were subsequently neutralized by the addition of HCl 0.1 M.

EVs and EV-Viruses were loaded with DiD lipohilic dye (EV-DiD) and were prepared by incubating 1 × 10^8^–5 × 10^9^ EVs for 1 hour at RT with 5 μL of DiD (Biotium, Rome, Italy) per mL of EV suspension in PBS. Next, the samples were centrifuged at 150,000× *g* for 3 hours to pellet the EVs. The supernatant containing unbound DiD was removed, and the EV-pellet was washed by suspending it in PBS and pelleting it again at 150,000× *g*. The final EV-DiD-Virus pellet was re-suspended in 100 μL of PBS and stored at −80 °C until use. EV-formulations were further characterized as previously reported [25].

### 2.3. MTS Cell Viability Assay

LL/2 cells were seeded at a density of 1 × 10^4^ cells/well in 96-well plates and maintained under appropriate conditions. On the following day cells were treated in triplicates with control EVs (10 virus-containing EV/cell), Virus (10 viral particle/cell) and EV-Virus (10 virus-containing EV/cell) as previously described [25]. Cell viability was determined by MTS assay kit according to the manufacturer’s protocol (Cell Titer 96 AQueous One Solution Cell Proliferation Assay; Promega, Nacka, Sweden). The absorbance was measured with a 96-well plate spectrophotometer Varioskan Flash Multimode Reader (Thermo Scientific, Waltham, MA, USA) at 490 nm. The experiments were independently performed three times with triplicates of each condition in each experiment.

### 2.4. Quantitative Real-Time PCR

qPCR for adenovirus E4 copy number was carried out according to the protocol previously described [28] (primer FW: 50-GGA GTG CGC CGA GAC AAC-30, primer RV: 50-ACT ACG TCC GGC GTT CCA T-30, probe E4: 50-(6FAM)-TGG CAT GAC ACT ACG ACC AAC ACG ATC T- (TAMRA)230). Total DNA was extracted from LL-2 cells 48 h post treatment in vitro using the QIAamp DNA Blood Mini Kit (Qiagen, Hilden, Germany) according to the manufacturer’s protocol. Subsequently isolated DNA was analyzed for adenoviral E4 copy number normalized to murine beta-actin (liver, blood) and human beta-actin (tumor), respectively (primer FW: 50-CGA GCG GTT CCG ATG C-30, primer RV: 50-TGG ATG CCA CAG GAT TCC AT-30, probe murine beta-actin: 50-(6FAM)-AGG CTC TTT TCC AGC CTT CCT TCT TGG-(TAMRA)230; (primer FW: 50-CAG CAG ATG TGG ATC AGC AAG-30, primer RV: 50-CTA GAA GCA TTT GCG GTG GAC-30, probe human beta-actin: 50-(6FAM)-AGG AGT ATG ACG CCG GCC CCT C-(TAMRA)230). Samples were analyzed using LighCycler qPCR machine (LighCycler 480, Roche, Basel, Switzerland).

### 2.5. Lung Tumor Model and Pharmacological Treatments

All the animal experiments were performed and approved by the Italian Ministry of Research and University (permission numbers: 12-12-30012012, 547/2015) and controlled by a Departmental panel of experts. C57BL/6 mice were used for the experiments; tumors were established by injecting 1 × 10^6^ LL/2 cells s.c. into the neck of 12-week old male mice. The following treatments were performed: Virus (*n* = 5) (1 × 10^8^ viral particles/injection) administered i.v., Virus (*n* = 5) (1 × 10^8^ viral particles/injection) administered i.p., EV-DiD-Virus (*n* = 5) (1 × 10^8^ virus-containing EV/injection (EV-V/injection)) administered i.v. and EV-DiD-Virus (*n* = 5) (1 × 10^8^ EV-V/injection) administered i.p. Treatment groups were injected with a volume of 100 µL to mice with tumors (one tumor per mouse about 5 mm in diameter).

### 2.6. In Vivo and Ex Vivo Imaging

Mice were anaesthetized using Isofluorane (Isofluorane-Vet; Merial, Lyon, France) and kept under anesthesia during imaging sessions carried out with the Imaging System (5 min for dorsal view and 5 min for ventral view) (IVIS Lumina II Quantitative Fluorescent and Bioluminescent Imaging; PerkinElmer, Waltham, MA, USA). Photon emission in selected body areas was measured using the Living Image Software 3.2 (PerkinElmer). For the *ex *vivo** imaging the mice were treated with luciferin 15 min prior to euthanasia by cervical dislocation and *ex vivo* imaging of the selected organs was carried out immediately after death. For the ex vivo imaging, acquisition of tissue explants was performed by 5 min exposure. Photon emission was quantified with the Living Image Software 3.2 (PerkinElmer). The ex vivo fluorescence imaging was carried out 24 hours post i.v. and i.p. EV treatments using IVIS Lumina II Quantitative Fluorescent Imaging (PerkinElmer, Waltham, MA, USA) with suitable filters (Cy5.5) and following the manufacturer instructions for fluorescence background subtraction. The quantification was done with Living Image Software 3.2 (PerkinElmer).

### 2.7. Immune Cell Infiltration Analysis

Immune cell infiltration of tumors was analyzed by flow cytometer BD LSR II (BD Biosciences, Milan, Italy) and FlowJo software (Tree Star, Ashland, OR, USA) at sacrifice (24 h post-treatment). Specific lymphocytes were quantified using antibodies CD45+ (Abcam, ab210185, Cambrige, MA, USA), CD3+ (Abcam, ab34275), CD4+ (Abcam, ab210348), CD8+ (Abcam, 25499). Tumors were harvested, weighted and dissociated with tumor dissociation kit mouse (Miltenyi Biotec 130-096-730, Bergisch Gladbach, Germany). Then, cells were washed and stained with antibodies according to the manufacturer’s instructions.

### 2.8. Statistical Analysis

Statistical significance was analyzed by using one-way ANOVA with Tukey’s Multiple Comparison test and nonparametric Mann–Whitney test. All statistical analysis, calculations and tests were performed using GraphPad Prism 5 (GraphPad Software, San Diego, CA, USA).

## 3. Results and Discussion

OV are immunotherapeutic agents with the potential to be used in combinatory therapies for advanced solid tumors refractory to current therapies [29,30]. The possibility of adopting a systemic route of administration and protection of the viral particles from the host immune attack remain critical issues that need to be addressed in order to extend the potential application of OV in cancer therapy. Systemic delivery of OV has been tested in a few clinical studies, where either the i.v. or the i.p. route of administration was chosen (ClinicalTrials.gov Identifier: NCT02053220) [31,32], (ClinicalTrials.gov Identifier: NCT02963831). The studies with the oncolytic adenovirus enadenotucirev (ColoAd1) and reovirus (Reolysin) for the treatment of different cancers gave feasibility data to support the use of i.v. infusion of the viruses (ClinicalTrials.gov: NCT02053220) [31,32], while i.p. injection was used in a Phase 1/2 study to investigate the safety, biologic and anti-tumor activity of the oncolytic adenovirus ONCOS-102 in combination with durvalumab in subjects with advanced peritoneal malignancies (ClinicalTrials.gov Identifier: NCT02963831).

**Figure 1:**
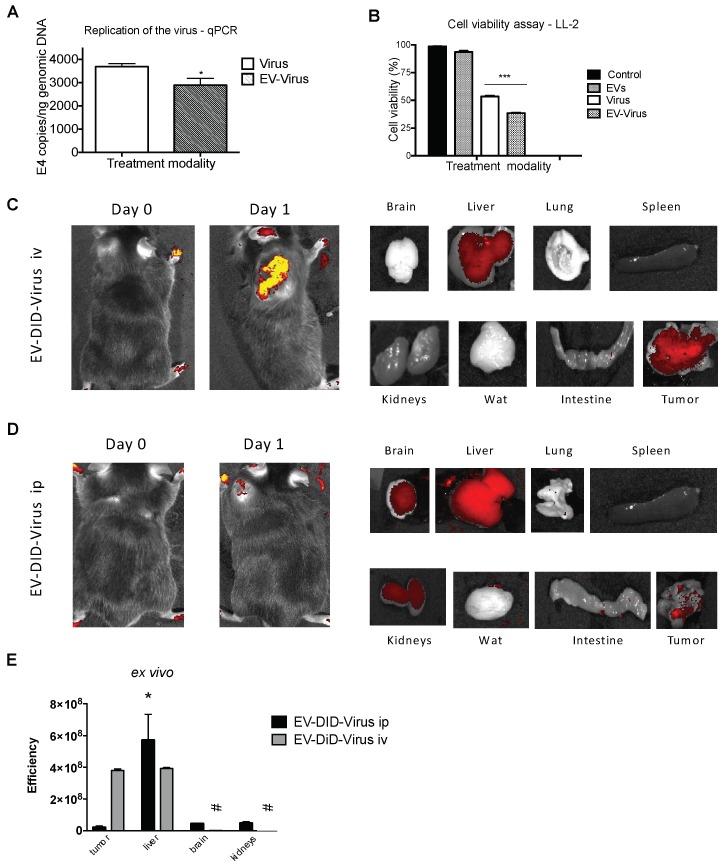
Extracellular vesicles loaded with oncolytic viruses show fluorescent signal in the tumor site after systemic injection. (**A**) Adenoviral copies towards E4 gene were analyzed by PCR from harvested LL-2 cell line after 24 h post-treatment. Error bars mean +/− SD, *p < 0.05. (**B**) Cell viability was determined against untreated cells (control), 72 h post-treatment. (**C**–**D**) Representative images that indicate the intensity of photon emission (fluorescence) in tumor area of C57BL/6 mice that were intravenously and intraperitoneally injected with EV-DiD-Virus (1 × 10^8^ EV-V/injection). (**E**) Quantification of fluorescence emission was assessed from resected organs using the Living Image Software (PerkinElmer) and CCD-camera (IVIS Lumina II Quantitative Fluorescent Imaging; PerkinElmer, Waltham, MA, USA). Efficiency is a fluorescence emission radiance per incident excitation power (p/s/cm^2^/sr)/(uW/cm^2^). **p < 0.05 vs EV-DiD-Virus ip tumor; ### *p* < 0.001 vs EV-DiD-Virus iv liver.

Due to their natural origin, EVs have recently had considerable attention as non-immunogenic drug delivery vehicles [17,18], and the i.v. or the i.p. route of administration was chosen in different *in vivo* studies [33,34,35,36]. In the current work, we protected the OV from immune disruption through their encapsulation into EVs (EV-Virus) [25] and tested the biodistribution of these particles when administered i.v. and i.p. in C57Bl/6 wild type mice bearing lung neoplasia generated by the s.c. injection of syngeneic LL2 lung cancer cells. The EVs were prepared as previously described and displayed biophysical characteristics, in terms of size distribution and Zeta-potential, very similar to those produced in our previous work [25]. The OVs encapsulated (EV-Virus) or not encapsulated in EVs (Virus) were tested for their ability to infect LL2 cells, even though these cells are of mouse origin. The results of the real time PCR and cell viability experiments demonstrated that indeed the virus was able to infect and induce cell death also in this mouse cell line (Figure 1A,B). To track the destiny of the injected EV-Virus within the body, we added the fluorescent dye DiIC18(5); 1,1′-dioctadecyl-3,3,3′,3′-tetramethylindodicarbocyanine and 4-chlorobenzenesulfonate salt (DID) [26], and measured the biodistribution of the particles by in vivo and ex vivo fluorescence imaging 24 h post-treatment (Figure 1C,D). In mice injected i.v. with the EV-Virus formulation, we observed a specific signal originating in the tumor area, suggesting that when directly injected into the systemic blood circulation, the particles were selectively homing into the neoplastic tissue (Figure 1C). This tumor-associated photon emission was not present in mice treated i.p. The ex vivo imaging analysis of the fluorescence emission from the dissected organs confirmed that the signal was mainly originating from the tumor in animals treated i.v. with only some photon emission arising from the liver (Figure 1C,E). In i.p.-treated mice, we observed a more generalized photon emission coming from liver, brain and kidneys, and only a minor emission from the tumor (Figure 1D,E). Thus, we concluded that i.v. administration of EV-DiD-Virus can more efficiently and selectively target the tumor tissue, compared to the i.p. route. The cancer-tropism of the EV-Virus formulation might be ascribed to the tumor-derived EVs, which are part of the cell-to-cell communication system recently characterized in neoplastic tissues [37]. It has been proposed that these vesicles have on their surface adhesion molecules selectively interacting with proteins present in the tumor membrane. When injected i.p. the EV-Virus loses this homing capability: we might speculate that the specific peritoneal environment modified the formulation, perhaps by inducing the release of the viruses from the vesicles, thus preventing the intact particles efficiently reaching the blood stream. The selective accumulation of the dye in the liver, brain and kidney upon i.p. injection with the EV-DiD-Virus might suggest that this route of administration could be useful for delivering the EV-Virus in these organs; future studies should address this possibility and directly prove the EV-Virus accumulation after i.p. treatment.

**Figure 2:**
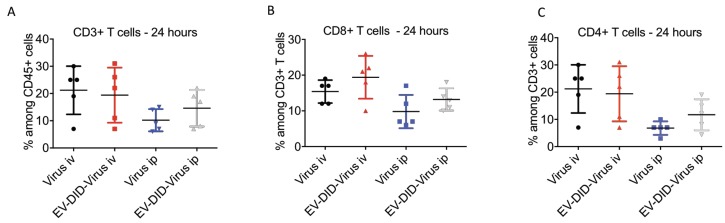
Tumor infiltrated lymphocytes induced by oncolytic viruses. (**A**–**C**) Percentage of murine CD45+, CD3+, CD4+, CD8+ T-cells was quantified from resected tumors by flow cytometer. Results are presented as mean +/− SD. *N* = 5/group. Error bars mean+/− SD. ANOVA one way analysis did not reveal statistical differences between the tested groups.

Although the LL2 cell lines are of mouse origin, as shown in Figure 1A, OVs demonstrated the ability to infect and kill these cancer cells *in vitro*. This prompted us to address further questions concerning the extent to which the procedure of encapsulation into EVs could modify the tumor-associated immune response raised in the host organism as a consequence of the viral infection. Immune modulation plays a critical role in the OV’s mechanism of action [38,39] and the presence of tumor infiltrating lymphocytes (TILs) is considered a hallmark of the anti-tumor immune response produced by the virus [38,39]. CD3+ and CD8+ T cell infiltration has been associated with a good prognosis [40] and improved outcome [41] in different cancers. Moreover, Ranki et al. reported a short-term, post-treatment increase of systemic pro-inflammatory cytokines and a marked infiltration of TILs in tumors in 11 out of 12 patients that responded to OV therapy [42]. To investigate the effects of EV encapsulation, we thus selected to measure CD3+ and CD8+ infiltration into the tumor as a parameter of the immune response [43], after i.v. and i.p. injections of the EV-Virus and Virus alone. Chemical inactivation of the free viral particles in EV-Virus formulations was performed as described in the Materials and Methods, to avoid their potential influence on the immune response evaluation. We found that the i.v. administration of EV-Virus resulted in CD3+, CD4+ and CD8+ T-cells infiltration in the tumor, comparable with that observed in animals i.v. injected with the Virus alone (Figure 2A–C). These results suggest that the procedure of EV encapsulation was not influencing the immune modulatory effects of the virus. In the tumors of mice that received the virus through i.p. injection, we observed a lower TIL infiltration (Figure 2A–C) in agreement with the reduced homing of the viral particles found at the tumor site (Figure 1D,E).

In conclusion, our results encourage the use of the i.v., rather than i.p., route of administration and suggest a procedure for the systemic delivery of OV based on their encapsulation into EV. This procedure could protect the viruses against their disruption by the host immune system, while efficiently targeting the therapeutic particles into the neoplastic tissue.
